# The Insulin-like Growth Factor Signaling Pathway in Breast Cancer: An Elusive Therapeutic Target

**DOI:** 10.3390/life12121992

**Published:** 2022-11-29

**Authors:** Ji-Sun Lee, Claire E. Tocheny, Leslie M. Shaw

**Affiliations:** Department of Molecular, Cell & Cancer Biology, University of Massachusetts Chan Medical School, 364 Plantation St., Worcester, MA 01605, USA

**Keywords:** insulin-like growth factor (IGF), IGF-1 receptor (IGF-1R), breast cancer, insulin receptor substrate (IRS) proteins, signal transduction, therapy

## Abstract

In this review, we provide an overview of the role of the insulin-like growth factor (IGF) signaling pathway in breast cancer and discuss its potential as a therapeutic target. The IGF pathway ligands, IGF-1 and IGF-2, and their receptors, primarily IGF-1R, are important for normal mammary gland biology, and dysregulation of their expression and function drives breast cancer risk and progression through activation of downstream signaling effectors, often in a subtype-dependent manner. The IGF signaling pathway has also been implicated in resistance to current therapeutic strategies, including ER and HER2 targeting drugs. Unfortunately, efforts to target IGF signaling for the treatment of breast cancer have been unsuccessful, due to a number of factors, most significantly the adverse effects of disrupting IGF signaling on normal glucose metabolism. We highlight here the recent discoveries that provide enthusiasm for continuing efforts to target IGF signaling for the treatment of breast cancer patients.

## 1. The IGF Signaling Axis

**Figure 1:**
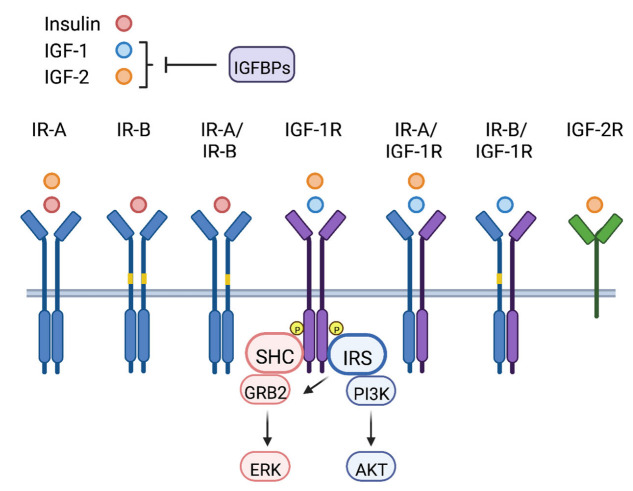
The insulin and IGF signaling (IIS) axis. The IIS pathway is comprised of ligands (Insulin, IGF-1, IGF-2), receptors (IR: insulin receptor; IGF-1R: IGF-1 receptor, IGF-2R: IGF-2 receptor), and IGF binding proteins (IGFBPs). IR and IGF-1R subunits homodimerize or heterodimerize to mediate downstream signaling through SHC and insulin receptor substrate (IRS) proteins. The IGF-2R serves as a decoy receptor to inhibit IGF-2 mediated signaling. Under normal physiologic conditions, insulin preferentially binds to the IRs, IGF-1 binds to the IGF-1R and IR/IGF-1R hybrid receptors, and IGF-2 binds to the IR-A, IGF-1R, and IR-A/IGF-1R hybrid receptors. IGFBPs control bioavailability of IGF-1 or IGF-2.

The insulin-like growth factors (IGFs) belong to the insulin and IGF signaling (IIS) pathway, which is comprised of a network of ligands, receptors, and binding proteins that mediate signaling to control and coordinate normal organismal growth and metabolism (Figure 1). IIS ligands include insulin, IGF-1 and IGF-2, and these peptides function as hormones and as local tissue growth factors (IGF-1 and IGF-2). IIS receptors include the insulin receptor (IR), IGF-1 receptor (IGF-1R) and IGF-2 receptor (IGF-2R). IR and IGF-1R are cell surface receptor tyrosine kinases (RTK) that share a high degree of homology (reviewed in [1]). Each of these receptors contain subunits of di-sulfide bonded extracellular α- and transmembrane β-chains that dimerize to form holoreceptors. The IR has two splice variants, IR-A and IR-B, that differ by the inclusion of exon 11 in IR-B that encodes a 12 amino acid region in the α-chain [2]. IR and IGF-1R subunits homodimerize and heterodimerize to form hybrid IR/IGF-1R receptors. The IGF-2R, also known as the mannose-6-phosphate receptor, is a single subunit transmembrane cell surface receptor [3].

Insulin, IGF-1 and IGF-2 each differ in their binding specificity and affinity for the IIS receptors (Figure 1). At physiological concentrations, insulin binds primarily to the IR-A and IR-B and IR-A/IR-B hybrid receptors, and IGF-1 to the IGF-1R and IR/IGF-1R hybrids [4]. IGF-2 binds to the IGF-1R and IR-A, as well as hybrid IGF-1R/IR-A receptors [2]. The 12 amino acid region encoded by exon 11 reduces the affinity of IR-B for IGF-2, making this ligand primarily selective for the IR-A isoform [2]. In addition to IR-A and IGF-1R, IGF-2 binds to the IGF-2R, which serves as a decoy or scavenger receptor to inhibit IGF-2-dependent signaling through the IR-A and IGF-1R [3,5]. The signaling potential of the IGF ligands is also impacted by a family of IGF binding proteins (IGFBP1-6) [6]. Although binding of the IGFBPs to IGF-1 and IGF-2 in the circulation stabilizes their expression, this interaction interferes with the binding of these ligands to their respective receptors, inhibiting their signaling activity. In this manner, the IGFBPs control the bioavailability of the systemic IGF ligands.

Upon stimulation by the IGF ligands, the IR and IGF-1R receptors undergo receptor trans-autophosphorylation by their intrinsic receptor tyrosine kinases [7]. Major signaling adaptors recruited to these phosphorylated receptors are the SHC and insulin receptor substrate (IRS) proteins, which upon phosphorylation by the receptors, initiate downstream signaling cascades to modify cellular outcomes (Figure 1) [4]. IRS-1 and IRS-2 are the most ubiquitously expressed IRS family members, and they are the primary mediators of insulin and IGF-dependent regulation of glucose metabolism and mitogenesis in most cell types (reviewed in [8]). Humans also express IRS-4, which is expressed in a more restricted manner, primarily in the kidney, thymus and liver [9]. An additional family member, Irs-3, is expressed in rodents but not in humans [10,11]. The major downstream signaling pathways activated by IIS, the ERK and PI3K pathways, are differentially dependent upon adaptor protein recruitment. Stimulation of the ERK pathway is primarily mediated through SHC, although the IRS proteins can also contribute to the activation of this kinase cascade. In contrast, PI3K recruitment and activation are dependent solely on the IRS proteins [12].

## 2. Physiological Functions of the IGF Signaling Pathway

In normal adult physiological contexts, IGF-1 stimulation of the IGF-1R regulates mitogenic signaling, whereas insulin stimulation of the IR, primarily IR-B, regulates glucose uptake to control organismal glucose homeostasis [13]. The mechanism of action of insulin and its importance for normal metabolic regulation, as well as its impact on breast cancer, have been reviewed extensively [14,15,16]. This review will focus on the physiological roles and mechanism of action of the IGF ligands and their involvement in breast cancer. The impact of targeting IGF-dependent signaling for breast cancer therapy on insulin-dependent regulation of metabolic homeostasis will also be discussed.

IGF-1 is expressed predominantly in the liver under control of growth hormone (GH) and released into the circulation where it travels to target tissues to bind IGF-1R for its growth regulatory action. Evidence for the essential role of IGF-1 signaling in normal regulation of growth comes from individuals with Laron dwarfism, a condition caused by deletion or mutation of the GH receptor that results in congenital IGF-1 deficiency [17]. The only treatment for patients with Laron Syndrome is recombinant IGF-1, which restores much of their growth deficits. *Igf1^−/−^* and *Igf1r^−/−^* mice are also small in body size, confirming the evolutionary importance of this growth regulatory pathway [18,19,20]. IGF-2 is also expressed primarily by the liver (fetal liver in rodents), and it contributes to embryonic growth [21,22,23]. This regulation is attributed to both its ability to signal through the IGF-1R and in part to stimulate IR-A, which is the predominant IR isoform expressed in fetal tissues [2]. In addition to acting as systemic hormones, IGF-1 and IGF-2 are also expressed locally in non-hepatic tissues where these ligands function in both an autocrine and paracrine manner to regulate tissue function [21,22].

The IRS proteins play important and distinct roles in the outcomes of IIS signaling in normal development and physiology, as highlighted by knockout mouse studies. *Irs1^−/−^* mice are born small and remain runted throughout their lives, similar to *Igf1^−/−^* and *Igf1r^−/−^* mice, implicating IRS-1 in somatic growth regulation [24,25]. In contrast, *Irs2^−/−^* mice are born normal in size but have small brains and are infertile [26,27]. Although both *Irs1^−/−^* and *Irs2^−/−^* mice exhibit insulin resistance, only *Irs2^−/−^* mice develop diabetes, emphasizing the key role of IRS-2 in metabolic regulation [26]. *Irs4*^−/−^ mice are phenotypically normal, with only mild insulin resistance and growth and reproductive defects [28], in keeping with the more restricted expression pattern of IRS-4. As will be discussed, the IRS proteins also contribute to disparate functional outcomes of IGF signaling in breast cancer.

The IIS pathway is tightly regulated under normal physiological conditions to control growth and metabolic homeostasis. Upon stimulation by ligands, receptors are internalized from the cell surface and their signaling longevity is controlled by endocytic trafficking either to the late endosome and lysosome for receptor/ligand degradation or to the recycling endosome for trafficking back to the cell surface for continued signaling [29]. The metabolic and mitogenic signaling differences of the IR and IGF-1R, respectively, may be explained in part by differential trafficking of each receptor and the longevity of their signals [30,31]. Signal duration is also impacted by negative feedback regulation of the IRS proteins through serine phosphorylation [32], which disrupts their interactions with upstream receptors and/or downstream effectors, or targets them for degradation via ubiquitin-mediated proteasomal degradation [33,34,35]. The consequence of this feedback is the cessation of signaling.

### The IGF Signaling Pathway and Normal Breast Biology

The IGF signaling pathway is essential for normal breast development and regulates function of the mature gland [36]. The number of terminal end buds (TEBs), the unique structure where the mammary gland stem cell niche is enriched, is significantly reduced in *Igf1*^−/−^ female mice, and TEB deficiency is rescued by administration of exogenous IGF-1 [37]. Studies with IGF-1 transgenic mice have also demonstrated that elevated circulating and local IGF-1 promotes mammary gland development [38,39,40]. IGF-2 is also essential for prolactin-induced mammary gland development [41,42]. In the adult mammary gland, IGF-1 and IGF-1R are required for alveolar differentiation during pregnancy and lactation [37,43,44].

The importance of the IGF signaling pathway for normal mammary gland biology also comes from crosstalk between this pathway and estrogen receptor-α (ER). Estrogen and ER are essential for normal mammary gland development and function. As a ligand-activated transcription factor, the activity of ER is mainly dependent on the bioavailability of estrogen. Upon binding to estrogen, ER homodimerizes and regulates gene transcription by binding to estrogen response elements (ERE) in the promoter regions of target genes [45]. While estrogen is the major regulator of ER signaling, other growth factors, including IGF-1, can impact ER signaling [46,47]. IGF-1, as well as insulin, at supraphysiological (1 > ug/mL) concentrations, enhance the activation of ER by estrogen, implicating the involvement of IGF-1R in maximal ER function [48,49]. IGF-1 can also activate ER in a ligand-independent manner through direct phosphorylation of ER by PI3K/AKT, mTOR, and MAPK signaling [50,51]. Crosstalk between the IGF pathway and ER signaling is also bi-directional. ER positively impacts IGF signaling by upregulating the expression of IGF-1R, IRS-1, and IGF-2 [52,53] and by downregulating the expression of IGFBP3 and IGF-2R [54,55]. Moreover, estrogen can stimulate the binding of ER to the IGF-1R to promote downstream signaling [56,57]. In summary, the ER and IGF signaling pathways interact at multiple levels, and in doing so they synergize to regulate normal mammary gland function.

## 3. The IGF Signaling Pathway in Breast Cancer

Expression of the ligands, receptors, and adaptor proteins of the IGF signaling pathway are altered in breast cancer, often in a subtype-dependent manner, leading to enhanced activity. These observations and the key role of IGF signaling in normal growth regulation have stimulated a considerable amount of interest in understanding the impact of IGF signaling on breast cancer risk, progression, prognosis, and the potential of this pathway as a therapeutic target.

### 3.1. IGF Signaling Pathway Expression in Breast Cancer

Breast cancer is categorized into five major subtypes by molecular and immunohistochemical profiling: luminal A (ER^+^PR^+^HER2^−^Ki67^low^), luminal B (ER^+^HER2^−^Ki67^high^ or PR^−^), luminal B-like (ER^+^HER2^+^), ERBB2/HER2-enriched (ER^−^PR^−^HER2^+^), and triple negative (TN)/basal-like (ER^−^PR^−^HER2^−^) [58,59]. Standard of care is dependent on molecular subtype and molecular signatures are used to develop new treatments. Thus, understanding the expression and function of the IGF signaling pathway in each subtype of breast cancer is important. Many studies have used genomic and transcriptomic datasets and immunohistochemical analysis of patient tumors to examine expression of the IGF pathway in breast cancer and its association with clinical outcomes [60,61,62,63,64]. Overall, IGF-1R expression is upregulated in breast tumors to a varying degree across subtypes, with higher expression observed in luminal A and B tumors than in HER2^+^ and TN tumors [63], an association that is likely related to the regulation of IGF-1R expression by ER. IGF-1R expression is only associated with prognosis in some breast cancer subtypes and the impact on outcome is varied. Higher levels of IGF-1R are associated with better prognosis in luminal B tumors, whereas they are associated with poorer prognosis in HER2-enriched tumors [64].

The IGF ligands are independently associated with increased risk and poor outcomes in breast cancer. Evidence for the association of IGF-1 with cancer risk overall is demonstrated by a reduced cancer incidence in Laron Syndrome patients [17]. A strong positive correlation between circulating levels of IGF-1 and breast cancer risk has been observed in many studies, specifically in premenopausal women [65,66,67,68,69]. Elevated expression of IGF-2 is also associated with increased risk of breast cancer development and has been implicated in disparities in breast cancer risk and survival outcomes in African American women [70,71]. An additional, important connection of the IGF ligands with cancer is the association of obesity with increased risk and poor outcomes in many cancer types, including breast cancer [72,73]. A major consequence of obesity is hyperinsulinemia, which drives both systemic and local production of IGF-1 and IGF-2 and increases IGF bioavailability by suppressing IGFBP expression [74]. IGF-2 expression is also increased locally in breast tumors through loss of imprinting, and its function is enhanced by loss of heterozygosity at the *IGF2R* locus. Loss of the IGF-2R allows IGF-2 to be more available for binding to and stimulating the IGF-1R.

Although total expression levels of the IGF-1R are variable among breast cancers and do not show a consistent association with survival outcomes, IGF signaling pathway activity is elevated in most breast tumors, as would be anticipated with increased ligand production. IGF-1R phosphorylation is observed across all breast cancer subtypes and is associated with poor patient survival. In a cohort of 438 patients, phosphorylated IGF-1R (pIGF-1R) was detected in 49.3% of tumors including TN, luminal, and HER2^+^/ER^−^ tumors, and pIGF-1R, but not total IGF-1R, was associated with poor survival at 15 years [75]. In a smaller cohort of 90 patients with invasive breast cancer, pIGF-1R expression was observed in >85% of all tumors spanning subtypes [76]. The caveat to these immunostaining studies is that phospho-antibodies used for the detection of pIGF-1R also recognize the phosphorylated IR. However, the importance of IGF-1R-specific functions in breast cancer is supported by the fact that an IGF-1-driven gene signature is associated with poor disease outcomes [77,78]. Together these studies indicate that IGF-1R pathway activity is a more accurate measure of function in tumors than relative receptor expression.

The IGF-1R localizes at the cell membrane, in the cytoplasm and within the nucleus in normal breast tissue and the intracellular localization of the IGF-1R has prognostic significance for breast cancer. In a nested case–control study of 312 women, cytoplasmic, but not membrane, IGF-1R staining in benign breast tissue was associated with higher risk of developing breast cancer [79]. As with total IGF-1R, associations between cytoplasmic IGF-1R localization and breast cancer prognosis are subtype dependent. Cytoplasmic IGF-1R is associated with longer disease-free survival (DFS) in ER^+^, but not ER^−^, tumors. In contrast, this expression pattern has an unfavorable prognostic impact on DFS in TN breast tumors [80], suggesting that subcellular localization of IGF-1R may play different roles in different breast cancer subtypes.

More recent observations suggest that nuclear IGF-1R may also play a significant role in breast cancer. Nuclear IGF-1R staining is associated with more aggressive tumors and poorer survival outcomes in many types of cancer [81]. IGF-1R nuclear translocation is closely associated with cell proliferation in multiple breast cancer cell lines and inhibiting IGF-1R nuclear localization reduces cell proliferation and migration in both non-malignant mammary cells and breast carcinoma cells [82,83]. These findings suggest that nuclear IGF-1R controls fundamental cellular processes in mammary tissue that are perturbed during malignant transformation. When in the nucleus, IGF-1R can directly bind DNA and regulate transcription [84,85]. Interestingly, IGF-1R binds to and stimulates its own promoter in the absence of ER expression in breast carcinoma cells, suggesting a unique role for the IGF-1R in ER^−^ breast tumors, although this has not yet been elucidated [86]. While emerging evidence suggests a role for nuclear IGF-1R in breast cancer, it remains to be seen whether IGF-1R within this subcellular compartment can serve as a prognostic marker in breast cancer.

The IRS proteins play essential roles in regulating the response of tumors to IGF signaling in breast cancer and the expression of these adaptor proteins in breast tumors also impacts patient outcomes. *IRS1* is an ER-regulated gene, and IRS-1 expression is highest in well-differentiated, ER^+^ luminal tumors and lowest in more poorly differentiated, higher-grade tumors that lack ER expression [87,88]. In contrast, IRS-2 is expressed at higher levels in ER^−^, basal/TN cells and tumors [89]. Similar to the IGF-1R, the localization of the IRS proteins also impacts function and prognosis. IRS-1 is localized in the cytoplasm or nucleus, while IRS-2 is excluded from the nucleus and is exclusively expressed in the cytoplasm or at the plasma membrane [90]. Nuclear IRS-1 is indicative of active ER signaling, as it interacts with ER to regulate gene expression [91]. It is not surprising, therefore, that expression of IRS-1 in the nucleus confers a better clinical prognosis, as it is associated with tumor sensitivity to therapies that target ER expression and function [92]. Membrane staining of IRS-2 is associated with decreased overall survival in breast cancer patients, in particular in patients with progesterone receptor (PR) negative tumors [90]. Recruitment of IRS-2 to the plasma membrane after IGF-1R activation may explain this observation. However, IRS-2 is recruited to and activated by additional surface receptors, including integrins, growth hormone receptor and cytokine receptors, suggesting that membrane IRS-2 staining in breast cancer could represent activity of IGF-1R-dependent and -independent pathways [12,93].

### 3.2. The IGF Signaling Pathway and Breast Cancer Initiation

Epidemiological studies support that the IGF pathway plays an important role in breast cancer risk, and a link between IGF signaling and breast cancer initiation is supported mechanistically by studies using IGF-1 transgenic mice. These mice exhibit an expansion of normal mammary stem and progenitor cells through symmetric cell division, and gene expression profiling of these cells reveals an increased propensity for transformation [94]. Similar to its role in regulating normal mammary stem cells, the IGF signaling pathway is also implicated in the regulation of breast cancer stem cells (CSCs). CSCs are a rare subpopulation of tumor cells with the potential to self-renew, evade proapoptotic signals and differentiate to restore tumor heterogeneity [95]. CSCs not only contribute to initial tumor development, but they are also implicated in secondary tumor growth and disease recurrence. CSCs in breast cancer are identified by cell surface markers such as CD133^+^, CD44^+^/CD24^−^ and CD29^+^, as well as by functional markers such as aldehyde dehydrogenase (ALDH) activity [96]. IGF-1R expression is elevated in CD44^+^CD24^−^ enriched cell populations, and IGF-1R^high^ cells exhibit greater mammosphere formation, a measure of stem cell activity, compared to IGF-1R^low^ cells [97]. Additionally, CSCs from human breast cancer xenografts exhibit increased IGF-1R phosphorylation, which indicates increased pathway activity [96,98]. Targeting IGF-1R preferentially decreases ALDH^+^ and CD44^+^/CD24^−^ CSC populations, suggesting that the IGF pathway sustains the CSC niche [96,98]. The fact that IR is not enriched in the CSC population [98], and that IGF-1 and IGF-2 are more effective regulators of CSC function than insulin, supports a dominant role for the IGF-1R in regulating breast cancer stemness [99].

While growing observations indicate the involvement of IGF pathway signaling in breast CSC regulation, the mechanism by which this pathway regulates CSC function is not as well known. The IRS proteins play key roles in determining functional outcomes downstream of the IGF-1R, and differential roles for the IRS proteins in breast CSC regulation have been reported. In ER^+^ breast carcinoma cells, IRS-1 interacts with phosphorylated PR, which enhances PR-mediated cancer stemness and resistance to endocrine therapy [100]. In contrast, in ER^-^ breast carcinoma cells, IRS-2 plays a dominant role in CSC regulation. IRS-2 dependent activation of PI3K stabilizes MYC expression through the inhibition of GSK3β activity. In doing so, IRS-2 dependent signaling sustains activation of MYC and MYC-dependent CSC regulation [99].

### 3.3. The IGF Signaling Pathway and Breast Cancer Progression

**Figure 2:**
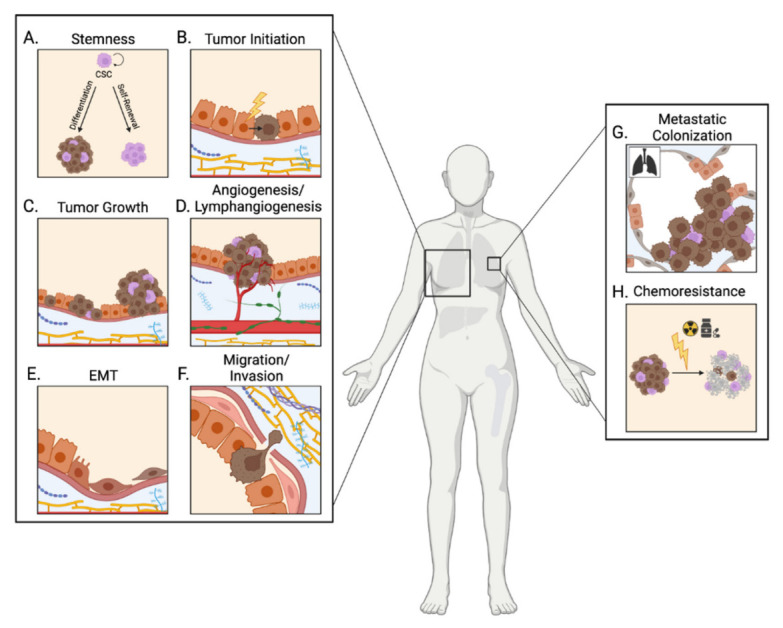
Functions of the IGF signaling pathway in breast cancer. IGF signaling is implicated at many stages of the multi-step process of breast cancer initiation and progression including: breast tumor initiation and cancer stem cell (CSC) regulation (**A**,**B**); primary tumor growth through the enhancement of proliferation and inhibition of apoptosis (**C**); promotion of neovascularization(angiogenesis) and lymphangiogenesis to facilitate tumor growth and dissemination (**D**); induction of epithelial-to mesenchymal transition (EMT) to enable tumor cell migration and invasion into the local tumor microenvironment (**E**,**F**); and metastatic colonization of secondary tissues (**G**). Hyperactive IGF signaling also confers resistance to a broad range of therapeutics (**H**).

A hallmark of breast cancer is the dissemination of primary tumor cells to secondary tissues. Tumor progression is a key prognostic factor for breast cancer patients, and aberrant activity of the IGF pathway has been implicated in several aspects of this complex, multistep process (Figure 2). This review will specifically focus on the involvement of IGF signaling in five key steps of breast tumor progression: epithelial-to-mesenchymal transition, local invasion, angiogenesis, secondary tumor formation, and chemoresistance.

*Epithelial-to-Mesenchymal Transition (EMT)*: Both in vitro and in vivo studies have connected IGF signaling with EMT, a multi-step developmental process that occurs in breast cancer in which cells adopt mesenchymal features, including increased motility and loss of cell–cell adherence junctions and polarity. Most breast tumors are carcinomas (tumors of epithelial origin), and breast tumors with poorer prognosis are those that adopt mesenchymal features [101,102]. Human breast carcinoma cell lines undergo EMT when treated with a variety of growth factors, including the IGF ligands [103]. Breast carcinoma cells stimulated with IGF-1 have decreased expression of the epithelial marker E-cadherin and increased expression of the mesenchymal marker vimentin and adopt a fibroblast-like, mesenchymal morphology [104]. Normal mammary epithelial cells expressing a constitutively active IGF-1R also adopt a mesenchymal morphology and express high levels of vimentin and additional mesenchymal markers N-cadherin and fibronectin, as well as low levels of E-cadherin [105]. Appropriate E-cadherin and mesenchymal marker expression is restored when these cells are treated with an IGF-1R inhibitor, suggesting that IGF-1R signaling is responsible for EMT-like changes. Cells expressing constitutively active IGF-1R also invade through Matrigel in vitro and form undifferentiated carcinomas in vivo, highlighting that IGF-1R-driven EMT correlates with tumor transformation and invasion. EMT is strongly driven by multiple signaling pathways, including NF-κB, PI3K/AKT, Wnt-1, and the MAPK family, that activate several transcription factors, namely ZEB1/2, Snail (SNAI1/2), Nanog, Oct-4, and Twist, and has been previously reviewed [106,107]. Activation of the PI3K and MAPK signaling pathways downstream of the IGF-1R is thought to regulate both ZEB1/2 and Snail1/2 to promote EMT [104], although activity of other kinases downstream of the IGF-1R, such as focal adhesion kinase (FAK) and Src, have also been implicated in this process [108,109].

Recent evidence suggests that the role of the IGF pathway in EMT is more complex than previously thought. In transgenic mice expressing a dominant negative IGF-1R (dnIGF-1R), tumors expressing Wnt-1 with diminished IGF-1R activity have increased expression of EMT mediators Twist-1 and Nanog [110]. Expression of IGF-2 is significantly increased in dnIGF-1R/Wnt-1 dual expressing tumors compared to Wnt-1 expressing tumors. Moreover, in IGF-1R null tumors, IGF-2 increases β-catenin levels, a key mediator of EMT. This effect is thought to be due to interactions between IGF-2 and the IR-A isoform, which, when combined with previous observations, suggests that IGF signaling can support EMT through both IGF-1R-dependent and independent mechanisms.

*Local Migration and Invasion*: Most breast cancers are invasive, and infiltration of cells from primary tumors into local breast tissue, vasculature, and lymphatics enables progression to metastatic disease. The migratory and invasive potential of tumor cells is mediated by a combination of intrinsic structural changes and extrinsic alterations in the surrounding extracellular matrix (ECM). In addition to loss of cell–cell adherence junctions, tumor cell migration requires the formation of membrane protrusions, namely lamellipodia and invadopodia, that extend into the ECM and, in combination with focal adhesions, direct cell movement [111]. IGF signaling is an important component of breast tumor cell motility. Breast carcinoma cell lines exhibit more aggressive, migratory behavior when stimulated with IGF-1 or when overexpressing IGF-1R in vitro [108,112], while knockdown of IGF-1R expression impairs colony formation and invasion [108]. IGF-1 promotes breast carcinoma cell migration through the formation of lamellipodia and focal adhesions [108,113,114]. Activation of multiple downstream signaling pathways, including both the PI3K and MAPK signaling pathways, as well as FAK and Src are required for IGF-1 dependent regulation of these structures [108,113,115].

Remodeling of the ECM is also necessary for invasion, as basement membranes and surrounding ECM components are physical barriers for tumor cells. To facilitate escape from this barrier, invading cells express a variety of proteolytic enzymes, including matrix metalloproteinases (MMP) and cathepsins, as well as urokinase plasminogen activator (uPA) to digest the matrix [116]. IGF-1 has been shown to upregulate the expression of MMP-9 and uPA, as well as MMP-9 activity, in breast carcinoma cells, though this regulation may be cell-type specific [117,118,119]. MMPs have also been shown to increase IGF bioavailability by digesting IGFBPs, which may perpetuate IGF-1R signaling [120,121]. The regulation of IGF-1R-mediated signaling by IGFBPs in breast cancer, however, is complex, and the role of MMPs in this context has not yet been fully elucidated [122,123].

Previous studies support that IGF-1R-driven migration and invasion is preferentially regulated through IRS-2. IRS-2 is highly expressed in invasive breast tumors, whereas IRS-1 is predominantly expressed in localized tumors [89], and rescue of IRS-2, but not IRS-1, expression in *IRS1/2* double null breast tumor cells restores invasive potential [124]. In addition, IGF-1 enhances IRS-2 phosphorylation to a greater degree than IRS-1 in metastatic mammary tumor cells, and knockdown of IRS-2 expression reduces IGF-mediated motility in vitro [125]. IRS-2-expressing breast carcinoma cells are less invasive after treatment with an IGF-1R/IR small molecule inhibitor, which emphasizes the importance of upstream IGF-1R signaling through IRS-2 for the regulation of invasion [124]. IRS-2 directs IGF-1R-dependent invasion via both PI3K-dependent and -independent mechanisms [124]. Processes downstream of IRS-2, but independent of PI3K, that promote breast cancer invasion have not yet been extensively investigated, and future studies examining these mechanisms may reveal novel approaches for targeting IGF-1R signaling in breast cancer.

*Neovascularization and Lymphangiogenesis*: Formation of new blood vessels provides local tumors with ample nutrients and oxygen for growth and provides access to the circulation. Low-oxygen environments in tumors prompt the activation and coordination of multiple pro-angiogenic processes [126]. IGF-1R, IGF-1, IGF-2 and IRS-2 synthesis is increased in multiple tissues by hypoxic conditions [127,128]. IGFs in turn can induce the expression of the transcription factor hypoxia inducible factor 1 alpha (HIF-1α) and its transcriptional target vascular endothelial growth factor (VEGF) [129], both of which are major regulators of new blood vessel growth. IGF-1 and IGF-2 both directly induce endothelial cell tube formation in vitro [130,131], suggesting that the IGF system can promote neovascularization through multiple mechanisms.

New lymphatic vessel growth also provides an avenue for breast cancer metastasis. Most breast cancers spread through the lymphatic system, and dissemination of tumor cells to regional lymph nodes is an important prognostic factor for breast cancer patients. IGF-1 can induce the production of VEGF-C, a key pro-lymphangiogenic factor that stimulates lymphatic endothelial cell proliferation and migration and has been shown to promote breast cancer metastasis [132,133]. IGFs also stimulate migration and proliferation of lymphatic endothelial cells in vitro [134], highlighting that the IGF system plays direct and indirect roles in establishing a peritumoral lymphatic network.

*Metastatic Colonization of Secondary Tissues*: Breast cancers typically metastasize to the lung, liver, brain, and bones, and the ability of tumors to colonize these organs depends on both growth and survival signals within the tissue microenvironment. IGF signaling is thought to enhance breast tumor growth within secondary organs by promoting proliferation and enhancing survival through apoptosis inhibition. Many studies have established that IGF-1 and IGF-2 increase proliferation and growth of breast carcinoma cells in vitro [135,136,137], and mouse models reveal that tumor growth in distal tissues is enhanced by signaling through the IGF-1R. In these studies, breast tumors expressing a dnIGF-1R that colonize the bone display reduced mitosis and increased apoptosis compared to tumors expressing functional IGF-1R [135]. Similarly, inhibition of IGF-1R function using picropodophyllin (PPP) reduces breast tumor proliferation in the brain [137]. Furthermore, breast tumor-bearing mice treated with a combination of IGF neutralizing antibodies and traditional chemotherapy have smaller lung metastases with fewer proliferating cells when compared to those treated with chemotherapy alone [76]. While proliferation significantly contributes to distal tumor growth, it is important to note that additional processes that the IGF pathway regulates in primary tumors, such as cancer stemness, EMT and invasion, also likely shape the successful establishment of secondary metastatic tumors.

Communication between secondary tumors and local tissues is key to proliferation and survival within a metastatic niche. Stromal cells are a major source of IGFs within the tumor microenvironment. In multiple in vivo breast tumor models, non-immune stromal cells and tumor-associated macrophages (TAM), but not tumor cells, primarily express *Igf1* and *Igf2* mRNA [76]. Cancer-associated fibroblasts (CAF), too, express *Igf1* RNA in tumors collected from a mouse model of invasive lobular carcinoma [138]. Notably, ex vivo studies have found that IGF ligands are also produced by brain pericytes and resorbed bone [135,137]. Breast tumor cells, and not stromal cells, are thought to predominantly express higher levels of IGF-1R. Together, these observations suggest that paracrine signaling between breast tumors and surrounding tissue, in which stromal-produced IGF ligands stimulate IGF-1R on tumor cells, is essential for secondary breast tumor expansion. IGF/IGF-1R signaling in distal tissues is likely not unidirectional, however, but rather a dynamic interplay between metastases and the local microenvironment.

*Chemotherapy Resistance*: Drug resistance significantly contributes to breast cancer progression and relapse and is often seen in patients with advanced disease. Studies have established that upregulation and overactivation of the IGF-1R confers resistance to many chemotherapeutic regimens, including hormone therapy, targeted agents, and cytotoxic antineoplastic agents. Breast carcinoma cells engineered to overexpress the IGF-1R become resistant to the ER targeting drugs tamoxifen and fulvestrant [139], and xenograft tumors derived from tamoxifen-resistant breast carcinoma cell lines express increased phosphorylated IGF-1R [140]. IGF-1R overexpression and activation has also been shown to negate the anti-proliferative effects of trastuzumab (HER2 targeted therapy), isoform-specific PI3K inhibitors, and radiation [141,142,143]. IGF-1R signaling decreases chemotherapeutic responses in breast cancer through multiple mechanisms, including promoting proliferation, enhancing cancer stemness, inhibiting apoptosis through DNA damage repair, and inducing efflux transporters [144,145]. Many clinical trials have tested the efficacy of IGF/IGF-1R inhibitors in improving responses to cytotoxic and hormone therapy in breast cancer patients. Although past studies have shown little measurable benefit, more recent trials have had promising results, and the potential clinical impact of targeting this pathway in treatment resistant breast cancer remains high.

## 4. Targeting the IGF Signaling Pathway in Breast Cancer

**Figure 3:**
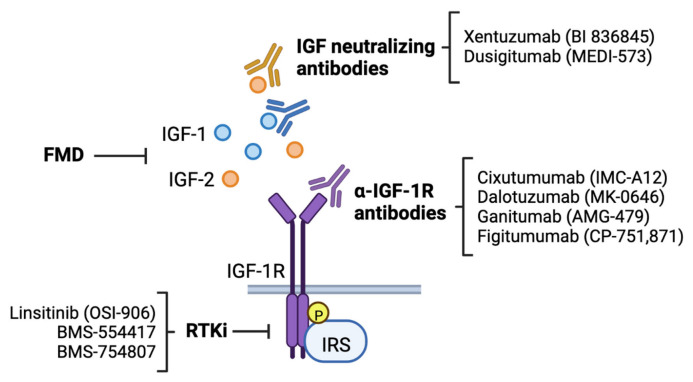
Therapeutics targeting the IGF signaling pathway. Anti-IGF-1R monoclonal antibodies block interaction between IGF-1R and the IGF ligands and induce internalization and degradation of surface receptors. Small molecule IGF-1R/IR receptor tyrosine kinase inhibitors (RTKi) block kinase activity to disrupt signaling. IGF neutralizing antibodies directly target IGF-1 and IGF-2 to prevent their interaction with receptors. Fasting mimicking diet (FMD) lowers basal glucose levels and suppresses IGF-1/IGF-2 expression and bioavailability. In doing so, FMD enhances the efficacy of chemotherapy.

Strong clinical associations between IGF-1R signaling and breast cancer risk and clinical outcomes, as well as extensive preclinical evidence implicating this pathway in multiple aspects of cancer progression, suggest a high therapeutic potential of targeting the IGF pathway in breast cancer. Substantial efforts have been made to produce anti-cancer therapeutics that target components of IGF-1 signaling (Table 1), which has resulted in the development of three major classes of inhibitors: anti-IGF-1R monoclonal antibodies, dual IR/IGF-1R tyrosine kinase inhibitors, and IGF neutralizing antibodies (Figure 3).

*Anti-IGF-1R monoclonal antibodies:* Monoclonal antibodies that selectively target the IGF-1R inhibit downstream signaling by blocking interactions between IGF-1R and its ligands and by decreasing surface expression of the receptor. Preclinical studies using both in vitro and in vivo model systems and Phase I clinical trials have shown that treatment with anti-IGF-1R antibodies can inhibit several steps in breast cancer progression, including proliferation, survival, motility, invasion, and metastasis [146,147,148,149,150]. As a result, multiple Phase II clinical trials were initiated to test the efficacy of several anti-IGF-1R antibody candidates, including IMC-A12 (cixutumumab, NCT00684983, NCT00699491), MK-0646 (dalotuzumab, NCT01605396, NCT00903006), AMG-479 (ganitumab, NCT00626106), and CP-751,871 (figitumumab, NCT00372996, NCT00976508), as a component of multidrug treatment regimens in both HER2^+^ and hormone receptor (HR) positive, HER2^−^ breast cancer. Unfortunately, results from Phase II trials have largely been negative, where treatment with anti-IGF-1R antibodies in combination with chemotherapy did not improve progression-free (PFS) or overall survival (OS) [151,152,153], and led to a variety of adverse side effects [154].

Poor efficacy of anti-IGF-1R antibodies may be due to compensation by signaling through the IR. IGF-1R inhibition commonly causes hyperglycemia, which induces endogenous insulin secretion. Hyperinsulinemia subsequently stimulates the IR to activate pathways capable of supporting breast tumor growth and progression. However, a recent Phase II clinical trial found that treatment with figitumumab in combination with the aromatase inhibitor exemestane may benefit breast cancer patients specifically with low HbA1c, a clinical indicator of non-elevated plasma glucose levels [155]. These results suggest that low glucose levels may be a predictor of patient responses to anti-IGF-1R antibodies, perhaps by minimizing secondary IR stimulation. Additionally, IGFs can bind both the IGF-1R and the IR due to structural homology between the receptors [156,157,158]. IGF-1R inhibition may therefore activate the IR pathway by shunting IGF-mediated signaling through the IR. Given the high degree of overlap between IGF-1R and IR signaling, including the formation of hybrid receptors, minimizing IR activation must be considered when developing strategies that improve effectiveness of anti-IGF-1R therapeutics.

*IGF-1R/IR Receptor Tyrosine Kinase Inhibitors (RTKi):* Small molecule inhibitors that target receptor tyrosine kinase activity of the IGF-1R (RTKi) have also been developed for use in breast cancer. Due to receptor homology, IGF-1R RTKi simultaneously target the IR, which limits IR signaling compensation seen with selective IGF-1R inhibition. Preclinical studies have shown that several dual IGF-1R/IR RTKi, including OSI-906 (linsitinib) [159], BMS-554417 [148], and BMS-754807 [160,161], exhibit anti-proliferative and pro-apoptotic affects in breast tumor cells, and inhibit breast tumor growth in vivo when given alone or in combination with hormone therapy. Phase I dose escalation trials of OSI-906 [162,163] and BMS-754807 [164] suggested that use of RTKi resulted in hyperglycemia but was ultimately tolerable and potentially had antitumor activity. Phase II trials were initiated to investigate the efficacy of BMS-754807 as a single agent and in combination with the aromatase inhibitor letrozole (NCT01225172) and OSI-906 in combination with hormone therapy and the EGFR tyrosine kinase inhibitor erlotinib (NCT01205685) but were ultimately terminated or withdrawn. Safety concerns were cited for study termination due to development of metabolic toxicities, indicating that approaches targeting the IGF-1R signaling system that preserve metabolic homeostasis are needed for the development of future anticancer therapeutics.

*Anti-IGF antibodies:* More recent approaches in targeting the IGF signaling pathway have focused on directly inhibiting IGF ligands. Dual IGF-1/2 neutralizing antibodies prevent IGF/IGF-1R/IR-A interactions without affecting receptor functions. Sequestration of IGFs has anti-proliferative effects in multiple cancer cell lines in vitro [165] and improves responses to traditional chemotherapy in a murine breast cancer model [76]. Importantly, hyperglycemia was not commonly seen in patients in Phase I dose escalation trials of IGF-1/2 neutralizing antibodies MEDI-573 (dusigitumab) [166,167] and BI 836845 (xentuzumab) [168], suggesting that anti-IGF-1/2 therapies may have a better metabolic safety profile compared to anti-IGF-1R antibodies or IGF-1R RTKi. Initial findings from larger clinical trials using anti-IGF neutralizing antibodies were discouraging, as a Phase Ib/II study of xentuzumab, exemestane, and everolimus (mTOR inhibitor) combination therapy for advanced HR^+^ breast cancer (NCT02123823) was discontinued early due to no change in PFS in the overall participant population [169]. However, further data analysis suggests benefit in patients without visceral metastases and led to the initiation of a new, follow-up Phase II trial (NCT03659136) that was recently completed. A Phase Ib/II trial investigating the efficacy of MEDI-573 with aromatase inhibitors for HR^+^HER2^−^ metastatic breast cancer (NCT01446159) has also recently been completed. Extensive results for these trials have yet to be released, and findings will shed light on potential future uses of these antineoplastic agents.

## 5. Future Directions

Antineoplastics targeting the IGF-1R and its ligands have largely disappointed in the clinic, despite extensive evidence that IGF signaling promotes many aspects of breast cancer progression. A significant hurdle to developing efficacious therapies is the disruption of normal glucose uptake into peripheral tissues, which increases plasma glucose and stimulates endogenous insulin and IGF-1 secretion. Future therapies that preserve normal glucose uptake and reduce peripheral glucose levels will prevent compensatory stimulation of the IGF-1R pathway in breast cancers.

Recent studies have investigated reducing glucose levels through diet (Figure 3). Fasting and low carbohydrate diets lower basal glucose levels and subsequent insulin and IGF-1 release and may increase the efficacy of therapeutics whose use decreases glucose uptake. Preclinical studies have shown that reducing environmental glucose in vitro and in vivo enhances effects of traditional chemotherapy. Breast carcinoma cells treated with a combination of doxorubicin, cyclophosphamide, and cisplatin exhibited increased DNA damage when cultured in low glucose conditions and mice harboring ER^+^ breast tumors were more responsive to hormone therapy when fed a fasting mimicking diet (FMD) diet than when fed an ad libitum diet [170,171]. Such promising results have brought dietary approaches to the forefront in recent clinical trials. A Phase II clinical trial evaluating impact of FMD on the response to neoadjuvant chemotherapy in HER2^−^ breast cancer (NCT02126449) found that complete or partial response to radiation more often occurred in patients fed a FMD than in patients who ate a regular diet [172], and a randomized Phase III trial to follow up these results has recently been registered (NCT05503108). Additionally, a Phase II trial investigating anti-tumor effects of FMD and metformin in TN breast cancer is currently recruiting patients (NCT04248998), highlighting an interest in restrictive diet as an approach to treat patients with multiple subtypes of breast cancer. One concern with the use of dietary therapies in breast cancer is the ability to maintain a restrictive diet for the entire duration of treatment. Nevertheless, results from these trials will inform the greater clinical community whether diet may be a viable therapeutic strategy, either alone or in conjunction with pre-existing IGF-1R-targeting therapies, for breast cancer patients.

Another potential, unexplored approach for inhibiting the IIS pathway in tumors is targeting the IRS proteins. The IRS proteins are commonly overexpressed in breast tumors, and in vitro knockdown of IRS expression limits breast tumor cell proliferation and invasive potential [124,173]. While inhibition of both IRS adaptors would likely alter metabolic signaling pathways, antineoplastic therapies targeting either IRS-1 or IRS-2 may preserve normal host metabolism. Targeting IRS-2, in particular, is intriguing as it is highly expressed in more aggressive breast cancers for which there are few effective treatments [89,125]. A recent study identified a unique region within the IRS-2 C-terminal tail that is necessary and sufficient for breast cancer cell invasion, but is not required for IRS-2 dependent regulation of glucose uptake [124], suggesting that the role(s) that IRS-2 plays in breast cancer progression can be separated from its role in glucose metabolism. Results from this study show that it is possible to selectively disrupt tumor-specific functions of the IRS proteins, and therapeutics that do so could inhibit IGF signaling without negatively impacting metabolic functions of normal tissues.

## 6. Conclusions

Epidemiological and mechanistic evidence supports an important role for the IGF signaling pathway in breast cancer. The disappointing results of efforts to date to target this pathway for the therapeutic benefit of breast cancer patients highlights the necessity of more research to better understand the molecular mechanisms by which this signaling pathway drives breast cancer progression. Identification of improved biomarkers that can identify responsive patient populations and new targets that are not essential for normal glucose homeostasis will provide a path forward to exploit this elusive target for clinical benefit.

## Figures and Tables

**Table 1:** Anti-cancer therapeutics targeting the IGF signaling pathway.

Therapeutic Class	Clinical Trial NCT Number	Phase	Treatment	Malignancy Type	Status	Reported Outcome(s)
IGF-1R monoclonal antibody	NCT00684983	II	cixutumumab (IMC-A12) + lapatinib + capecitabine	HER2^+^ Stage IIIB/VI breast cancer	Completed	no improvement in PFS, ORR, or OS
	NCT00699491	I/II	cixutumumab (IMC-A12) + temsirolimus	Male breast cancer; Recurrent breast cancer; Stage IV breast bancer	Completed	
	NCT01605396	II	dalotuzumab (MK-0646) + ridaforolimus + exemestane	High proliferation, ER^+^ breast cancer	Completed	no improvement in PFS
	NCT00903006	I/II	dalotuzumab (MK-0646) + fulvestrant + dasatinib	Metastatic HR^+^, HER2^−^ breast cancer	Terminated (low accrual)	
	NCT00626106	II	ganitumab (AMG-479) with and without exemestane or fulvestrant	HR^+^ locally advanced or metastatic breast cancer previously treated with endocrine therapy	Completed	no improvement in PFS; worse OS
	NCT00372996	II	figitumumab (CP-751,871) + exemestane	HR^+^ advanced breast cancer	Terminated (business reasons)	
	NCT00976508	I	figitumumab (CP-751, 871) + pegvisomant	Advanced solid tumors (colorectal, lung, breast, prostate, sarcomas)	Terminated (business reasons/low accrual)	
Dual IGF-1R/IR RTKi	NCT01225172	II	BMS-754807 with or without letrozole	HR^+^ breast cancer resistant to non-steroidal aromatase inhibitors	Terminated (business reasons)	
	NCT01205685	II	linsitinib (OSI-906) + letrozole with or without erlotinib	Hormone-sensitive metastatic breast cancer	Terminated (therapy toxicities)	
IGF-1/2 neutralizing antibody	NCT02123823	Ib/II	xentuzumab (BI 836845) + everolimus + exemestane	ER^+^ metastatic breast cancer	Completed	no improvement in PFS in overall population; evidence of benefit in patients without visceral metastases
	NCT03659136	II	xentuzumab (BI 836845) + everolimus + exemestane	HR^+^, HER2^−^ metastatic breast cancer without visceral disease	Completed	
	NCT01446159	Ib/II	dusigitumab (MEDI-573) with or without aromatase inhibitors	HR^+^, HER2^−^ metastatic breast cancer	Completed	
Fasting Mimicking Diet (FMD)	NCT02126449	II/III	neoadjuvant chemotherapy with or without FMD	HER2^-^ breast cancer	Completed	complete or partial response to radiation occurred more frequently with FMD
	NCT05503108	III	neoadjuvant chemotherapy with or without FMD	HR^+^, HER2^-^ metastatic breast cancer	Not yet recruiting	TBD
	NCT04248998	II	preoperative chemotherapy + FMD with or without metformin	TN breast cancer	Recruiting	TBD

ER = Estrogen Receptor; HR = Hormone Receptor; TN = Triple Negative; PFS = progression-free survival; ORR = objective response rate; OS = overall survival; RTKi = receptor tyrosine kinase inhibitor; FMD = fasting mimicking diet; TBD = to be determined.

## Data Availability

Our study did not report any data.
